# Use of amniotic membrane in bullous keratopathy palliative care

**Published:** 2014

**Authors:** GI Stefaniu, SM Chiotoroiu, FA Secureanu, VL Purcarea, M Zemba

**Affiliations:** * Clinical Ophthalmology Emergency Hospital Bucharest; ** ’’Nicolae Malaxa’’ Hospital, Bucharest; *** “Carol Davila” University of Medicine and Pharmacy, Bucharest; **** Department of Clinical Ophthalmology, “Dr. Carol Davila’’ Central Military Emergency Hospital, Bucharest

**Keywords:** bullous keratopathy, amniotic membrane, transplant

## Abstract

**Background:** Assessment of efficiency of amniotic membrane covering in the improvement of bullous keratopathy symptoms.

**Material and method:** The paper represents a clinical prospective study, which includes 42 patients diagnosed with bullous keratopathy and operated between January 2009 and November 2013 in the Department of Clinical Ophthalmology from “Dr. Carol Davila” Central Military Emergency Hospital. Follow up between 6 and 48 months, with an average of 22 months. Subject to research: corneal re-epithelisation, epithelial bullae, pain and photophobia relapse.

**Results:** In 37 cases, the symptoms improved, in 8 cases the minimum symptoms persisted and in 29 cases the symptoms completely disappeared. In 5 cases, there were no significant improvements, symptoms reappeared briefly after membrane resorption.

**Conclusions:** Amniotic membrane covering represents an efficient palliative care means in oedematous keratopathy.

## Introduction

Chronic corneal oedema may have multiple causes; two of the most frequent are Fuchs endothelial dystrophy and corneal endothelium trauma, mostly after intraocular surgery, usually during cataract surgery [**2**].

From the physiopathology perspective, oedematous keratopathy occurs due to an imbalance between forces pressing vitreous humour towards the cornea (intraocular and osmotic pressure of the stroma) and forces that remove water from the cornea (endothelial cells pump system). The decrease of endothelial activity level below the capacity to maintain cornea transparency can be temporary (generally, after intraocular surgery, when the post-operation trauma disrupts the endothelial cells’ activity, but most of the times the remaining cells can substitute the activity of the lost ones), either definitive (when the loss of endothelial cells can no longer be compensated by the remaining cells - in aggravated Fuchs dystrophy or in major surgical trauma) [**1**].

**Work assumptions**

Generally, it is thought that endothelial cells density below 500 cells per square millimetre cannot maintain corneal transparency. As for the concerns quantity, the quality of the endothelial cells is also important; sometimes a relatively high number of cells - 8-900 cells per square millimetre can associate with corneal oedema if such cells have important morphological and functional alterations.

Once the endothelium is no longer able to work as a pump, the stroma progressively accumulates fluid, thickening and developing plies on Descemet's membrane level. The next phase is that of epithelial oedema, occurring by fluid accumulation between the epithelial cells. The progression of the epithelial oedema results in the development of the epithelial bullae, rupture and cause which disturb the symptoms of oedematous keratopathy: pain, tearing, photophobia [**1**].

This affection is treated by transplant of cornea, in two versions: penetrating keratoplasty, when all the layers of the cornea and the posterior lamellar keratoplasty, or when only the corneal endothelium is replaced. Unfortunately, sometimes, the patients must wait for a long time until the transplantation can be performed, other times such an intervention is not recommended. For this reason, several types of interventions were attempted in order to improve symptoms: anterior stromal puncture, conjunctival covering, radial keratotomy, therapeutic photo-keratectomy by using the excimer laser [**3**,**7**], corneal cross-linking [**4**], amniotic membrane covering [**9**].

**Scope of research**

An efficient evaluation of amniotic membrane covering in the improvement of oedematous keratopathy symptoms: pain, tearing, photophobia.

## Materials and Methods

**Fig. 1:**
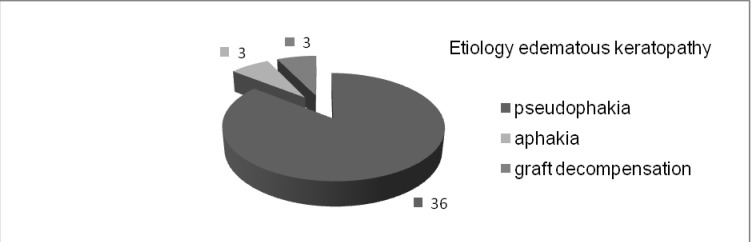
The etiology of edematous keratopathy

The paper represents a clinical prospective study within a period of 4 years. The study was conducted on a group of 42 patients operated by the same surgeon between January 2009 and November 2013. Only postoperative oedematous keratopathy cases were enrolled in the study, which occurred after the surgical intervention for cataract. As seen in **Fig. 1**, also 36 cases showed pseudophakia (28 posterior chamber, 6 anterior chamber, 2 of iris-claw type), 3 cases with surgical aphakia and 3 with penetrating keratoplasty and graft decompensation. In all the cases, visual prognosis was reserved. The minimum period of time between the settlement of oedematous keratopathy and surgical intervention was of 6 months, but most of the patients were diagnosed more than one year before that moment.

Amniotic membrane was sampled from pregnant women who gave birth by caesarean operation; they were tested for HIV 1 and 2, Hepatitis B, C, and syphilis at the beginning of the pregnancy, and were re-tested before the operation. All tests came out negative. The amniotic membrane was divided in small pieces (for single use) and frozen to - 20 degrees Celsius.

The surgical technique was the following: the operation was performed with retrobulbar anaesthesia; damaged epithelium was removed by using 90 degrees alcohol; a few drops of adrenalin 1% were introduced to diminish any potential bleeding during membrane suture. The membrane was placed with an avascular stroma towards the cornea and the epithelium [**5**]. The amniotic membrane was sutured with 10.0 sutures directly on the subjacent conjunctiva, 2-3 millimetres from the limb; for half of the sutures were put through the episclera for a better and firmer fixing of the membrane. In some cases, the conjunctiva was peeled off the limb and the membrane was sutured to the edge of the conjunctiva, but the blood from the conjunctival incision entered underneath the membrane, making the intervention more difficult. Postoperative treatment consisted in antibiotics and on-steroidal anti-inflammatory drugs, as well as artificial tears, gel, for 2 weeks. In most of the patients, the membrane was resorbed between 10 and 14 days. Sutures were removed after 14 days, during the consultation.

The patients were examined in the first and second day after the operation, after that they were released from the hospital and consultations were performed: first 1-2 weeks after, and then at every month, every 3 months, 6 months and then annually. The follow up period varied between 6 and 42 months, with an average of 22 months.

## Results

Results were very good on short term. During the 1-2 weeks consultation, no patient had epithelial bullae and none of them had any pain or photophobia. Once the amniotic membrane was resorbed and the mechanic protective effect disappeared, the symptoms reappeared. One month after, two of the cases had intense pain and showed numerous epithelial bullae and disepithelisation, and in two of the cases, ocular pain and photophobia reappeared, but intermittently, bearable as intensity and with significant improvement through medication. Another case had intense pain 3 months after surgery, epithelial bullae and deep corneal ulcer, which took a negative turn, in spite of the treatment, and required evisceration; moreover, there was another case with intense pain and photophobia and in four cases, the symptoms were acceptably intense. In the interval of 3-6 months, another patient with unfavourable eye evolution chose evisceration. After six months of follow up, two cases needed evisceration; three patients showed no improvement, in six cases symptoms partially disappeared and in thirty-one of the cases symptoms completely disappeared. Between 6-12 months of follow up, another two cases showed a bearable degree of pain and photophobia, relieved in one case with medication and in the other by applying therapeutic contact lens. Between 12-48 months, no other cases with disturbing symptoms due to corneal oedema appeared.

**Fig. 2:**
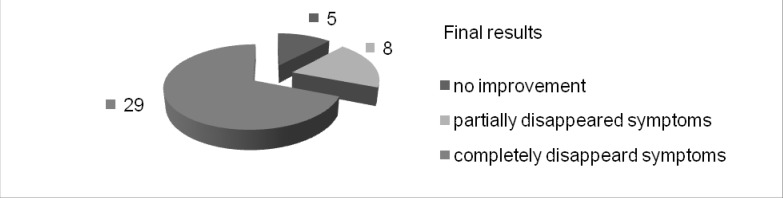
Final results

The final results as seen in **Fig. 2** were the following: five cases showed no improvement, in eight cases the symptoms partially disappeared and in twenty nine cases the symptoms completely disappeared.

## Discussions

The amniotic membrane is composed of an avascular stroma and a thick basal membrane, formed mainly of collagen and laminin.

The main properties of the basal membrane are: to improve adhesion of epithelial cells, to facilitate the migration of epithelial cells, to prevent apoptosis of the same epithelial cells. Also, the amniotic membrane contains specific growth factors (epithelial growth factor-EGF, keratinocyte growth factor-KGF), with an additional re-epithelisation effect. Due to its capacity to inhibit the beta growth and transformation factor-TGF-beta amniotic membrane reduces stromal cicatrisation, capacity which is less useful in our case and more when the amniotic membrane is used to treat the corneal burns [**7**,**8**]. An improvement of the patients’ symptoms for 37 patients, which represent 88%, was received as a result of the research. It is true that for some of them the improvement was only partial, turning from unbearable or hardly bearable to acceptable. The disappearance of the disturbing symptoms except, of course, for poor visual acuity, which maintained for all the patients, it was obtained only for 29 of the patients, meaning 69%. The results were relative; the number of patients subjected to follow up decreased along with the increase of the follow up period. It is possible that some of these cases have chosen to be treated by other physicians, because symptoms reappeared; that is why the results were presented after one year of follow up. The results we obtained are similar to the ones in literature. Espana obtained, as a result of a study conducted on 18 eyes, performed at Bascom Palmer Eye Institute di Florida, an improvement for 88% of the patients in 6 months [**5**]. At Bascom Palmer Eye Institute, Pires coordinated a multicentre study (5 centres) on 50 eyes and obtained a symptom improvement on 43 eyes [**6**]. The reasons for failure have also been studied. The first element considered was the type of artificial eye lens; of the 5 cases with completely unfavourable evolution, 3 were anterior chamber pseudophakic, one on the posterior chamber and one was aphakic. Also, in partial symptoms remittent cases, 2 had anterior chamber eye lens, two had postoperative aphakia, and one had penetrating keratoplasty and 3 posterior chamber eye lens. Practically, of the 6 cases of anterior chamber eye lens, 5 had some degree of symptoms maintenance. It is possible that the presence of the eye lens in the anterior chamber, even if is not the cause of oedematous keratopathy, but the postoperative trauma and the more difficult operation which, in the end, needed the implantation of this type of eye lens, to have a negative influence on the evolution of the cornea, in time. Another common element in all the cases, that was unresponsive to treatment and which was present in 6 of the 8 cases with partial response, was refractory secondary glaucoma. This element may explain the failure of such therapeutic intervention. It is possible that high intraocular pressure exercises much too high pressure so that the corneal epithelium, even if structurally improved, would resist without forming epithelial bullae, with characteristic symptoms.

## Conclusions

Amniotic membrane transplantation is an efficient palliative method for the patients with oedematous keratopathy with reserved visual potential. An improvement of symptoms was acquired in 88% of the cases, though only partial for 19%. The presence of an anterior chamber eye lens seems to be a negative element in oedematous keratopathy evolution. Of the cases with unfavourable evolution, 11 out of 13 (84%) also presented secondary glaucoma.
